# 16S rDNA based skin microbiome data of healthy individuals and leprosy patients from India

**DOI:** 10.1038/s41597-019-0232-1

**Published:** 2019-10-22

**Authors:** Nitin Bayal, Sunil Nagpal, Mohammed Monzoorul Haque, Milind S. Patole, Vijayalakshmi Valluri, Rohini Suryavanshi, Sharmila S. Mande, Shekhar C. Mande

**Affiliations:** 1 National Centre for Cell Science, NCCS Complex, Pune, India; 2 BioSciences R&D, TCS Research, Tata Consultancy Services, Pune, India; 3 Immunology & Molecular Biology Department, Bhagwan Mahavir Medical Research Centre, A. C. Guards, Hyderabad, Telangana State India; 4 Richardson Leprosy Hospital (The Leprosy Mission Trust), Miraj, Maharashtra India; 5 Council of Scientific & Industrial Research, Anusandhan Bhawan, 2, Rafi Marg, New Delhi, India

**Keywords:** Microbiome, Pathogens, Infectious diseases, DNA sequencing

## Abstract

Leprosy is an infectious disease that has predilection in skin and peripheral nerves. Skin has its own microbiome, however it is not extensively studied in Indian leprosy patients. Here, by using next-generation 16S rDNA sequencing, we have attempted to assess the skin associated microbial diversity pertaining to affected and unaffected skin of Indian leprosy patients. A total of 90 skin swab samples were collected from 60 individuals (30 healthy controls, 30 patients) residing in Hyderabad and Miraj, two distinct geographical locations in India to assess the homo/heterogeneity of skin microbial signatures. While a large increase in genus *Methylobacterium* and *Pseudomonas* was seen in patients from Miraj and Hyderabad respectively, a considerable decrease in genus *Staphylococcus* in the leprosy patients (as compared to controls) from both geographical locations was also observed. We expect that, these datasets can not-only provide further interesting insights, but will also help to observe dynamics of microbiome in the diseased state and generate hypotheses to test for skin microbiome transplantation studies in leprosy.

## Abstract (summary)

**Table:** 

Measurement(s)	DNA
Technology Type(s)	DNA sequencing
Factor Type(s)	experimental condition
Sample Characteristic - Organism	Homo sapiens
Sample Characteristic - Environment	microbial community
Sample Characteristic - Location	India

Machine-accessible metadata file describing the reported data: 10.6084/m9.figshare.9897089

## Background and Summary

Microbiome and dermatology research are related to each other^1–3^. Microorganisms generally thrive as site-specific close knit communities, however, at species and strain level, they can be as unique as human fingerprints^1^. Skin is the largest organ of human body and one of the most complex and unexplored areas in microbiome investigation^2–6^. High-throughput sequencing technologies have become the main stay of modern scientific studies in microbiome space^7,8^. Particularly, 16S ribosomal RNA gene analysis is considered as a gold standard for taxonomic identification of bacteria, also referred to as meta-taxonomic analysis^9,10^.

The morphology and physiological characteristics of various sites on the human skin define the composition of microbial communities^11,12^. Besides serving as a physical barrier that resists infiltration by potential pathogenic bacteria, skin microbiome is known to play diverse physiological roles in maturation and homeostasis of cutaneous immunity^13,14^. In cutaneous biology, either a shift in microbiome, genetic predisposition, misuse of antibiotics or loss of beneficial organisms is believed to aggravate the inflammatory skin disease conditions^15^. This is known as dysbiosis, which leads to an aberrant activation of immune system, resulting in compromised regulation of immune responses to commensal microbes^16^. There are various dermatological conditions that have visual and technical differences in their symptoms and diagnosis^17,18^.

Leprosy is one of the serious skin disorders found to have been linked to dysbiosis^19^. It is a chronic systemic granulomatous disease that primarily affects the skin and peripheral nerves, and less commonly mucosa of the upper respiratory tract, testes and the anterior chamber of the eyes^20^. It is caused by bacteria namely, *Mycobacterium leprae*^21^ and *Mycobacterium lepromatosis*^22,23^. If left untreated, leprosy can cause irreversible damage^23^ to its manifested body parts, which is one of the reasons for the attached social stigma^24,25^. Ridley and Jopling classified leprosy in a variety of clinical forms viz. tuberculoid (TT/ paucibacillary), borderline (BL), or lepromatous (i.e multibacillary LL/MB) based on the immune response to the organism^26^.

Studies on Acne^27,28^, Vitiligo^29^, Atopic dermatitis^30,31^ and Psoriasis^32^ have shown that these dermatological conditions exert influence on commensal microbiome and exhibit compositional variation in the disease onset or progression at the lesional site and at the systemic level. The extent to which skin microbiota changes after a systemic antibiotic intervention depends on the chemical nature of the antibiotic, combinations of antibiotics, type of administration, duration and dose. Although skin is physically compartmentalized from other body sites, cross-inoculation can lead to spread of antibiotic resistant microorganisms to skin^33^.

A key study involving Brazilian leprosy patients provides an in-depth analysis of the composition of skin microbiome in lepromatous, skin lesions (and paired adjoining non-lesional areas) sampled from a Brazilian patient cohort^34^. Results of this study primarily indicate that both lesional as well as non-lesional skin of (treated/untreated) individuals infected with leprosy harbors microbial communities that are significantly less diverse as compared to the skin of healthy individuals. This reduced diversity could possibly be attributed to the impact of the infectious agent (*M. leprae*) itself or it could be a systemic change resulting due to the ongoing MDT regime. Furthermore, there is a strong association between the lung microbiome and tubercle formation in an infection caused by *Mycobacterium tuberculsosis*^35^. Similarly whether a similar relationship exists between skin microbiome and progression of leprosy needs further studies.

It is of interest to study and contemplate the relationship between dysbiosis of skin microbiota and its effect on host skin metabolism and outcome of the leprosy. Given this context, we endeavored to study the structure of skin microbiome in healthy controls as well as of leprosy patients from India, a country that accounts for the highest annual number of leprosy cases diagnosed/reported worldwide. In 2016, 34,000 new cases were reported alone from endemic states in India with 25% annual cases^36^. Given high variability with respect to dietary habits, climatic and life-style conditions within India, we chose two well-separated geographical locations within India (viz. Lepra Blue Peter Public Health and Research Centre, Hyderabad and TLM Richardson Leprosy Hospital, Miraj) to check if there are differences created as a result of such potential confounding factors. These centres are specialized in diagnosis, treatment and management of leprosy including studies in related epidemiology.

This is the first study of dysbiosis of skin microbiome in leprosy patients as compared to healthy individuals from India. We report data from 60 study participants including 30 healthy individuals and 30 patients who were undergoing oral multi-drug treatment (MDT) therapy. The subsequent sections of this report provide details pertaining to study design, sample collection methodology, types of metadata collected, bioinformatics methods used for obtaining taxonomic abundance tables corresponding to sequence data, and the types of data that are being provided along with this report for public use.

## Methods

### Study design

**Fig. 1:**
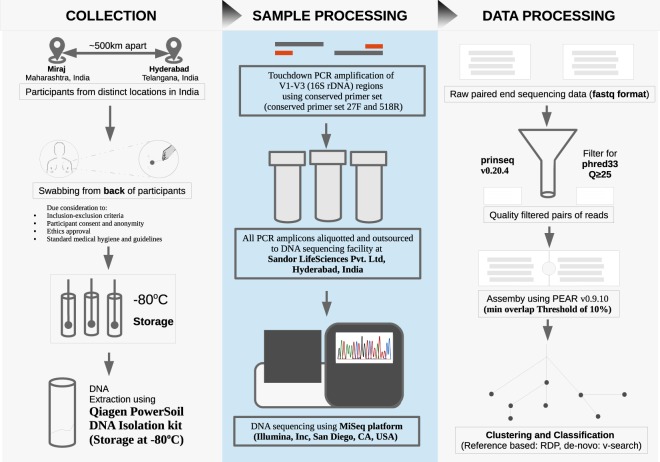
Schematic workflow depicting overall study design and methodology followed for sample collection, processing and data processing.

Figure 1 depicts the overall design of the present study. The present study was primarily an observational; non-interventional study conducted at two distinct centers viz. Hyderabad and Miraj (i.e. two Indian cities approximately 500 kilometers apart). The Institutional Ethical Committees of National Centre for Cell Science (NCCS), Pune as well as the respective sampling locations approved the study (IEC Approval Ref. No. NCCS/IEC/2016-I/1). The collection protocol was developed in line with the Nuremberg Code^37^ and Declaration of Helsinki^38^. Personal identifiable information pertaining to all participants was removed to ensure confidentiality^39,40^. All participants were provided a detailed explanation of study objectives and the sampling procedure, and written consent documents were obtained. Cohort size for the study was not statistically derived. The following inclusion and exclusion criteria were used for selection of consenting study participants (who were diagnosed for leprosy disease).

### Inclusion criteria

1. Age between 21 to 70 years.
2. Patients having a high (3+) bacillary index (MB/BL/LL).
3. Active patches present on the patients back (scapular and lumbar regions).

### Exclusion criteria

1. Pregnant women.
2. Patients co-infected with Leprosy-TB or Leprosy-HIV.
3. Individuals with a different illness or those who have taken any medications in previous 10 days for any other symptoms.

### Sample collection process

Person collecting samples mandatorily wore a sterilized clean lab apron, double layered gloves, hair cap and laboratory facemask to avoid any sort of cross-contamination. Sampling was done by researchers in a dedicated sample collection room. A fresh pair of gloves was used while collecting samples from every new participant of this study. As the aim of the study was to study skin microbiome, a non-invasive method of collection of skin swabs was performed instead of skin scrapings or skin biopsy^41,42^. Individual’s comfort was also taken in priority while sampling. A swabbing procedure was employed wherein two samples (5 cm × 5 cm) were collected from an affected lesional area and an adjoining uninfected portion (5–8 cms away from the lesional site). The skin swabs were collected from back area of the body using HiCulture Sterile Skin Swab collection device (HiMedia Labs, India) soaked in wetting solution (0.15 M NaCl with 0.1% Tween 20). The swabbing technique was executed as follows: Skin areas were selected and stretched with one hand, the swab in the other hand was held in such a manner that that the swab shaft is parallel to skin surface. Subsequently, the swab was moved in one direction for fifty times, thrice facing each side of the swab applying firm pressure. The collected swab samples were stored in −80 °C until further processing. Section A of Data Record titled, ‘Sample Collection Image’ depicts an image of protocol followed for skin swab sampling and Section B represents an image for a lesional site of one of a leprosy patient suffering from lepromatous leprosy as per records.

### DNA isolation and processing

DNA extraction was carried out using Qiagen PowerSoil DNA Isolation kit protocol with indicated modifications. Each swab was cut and swirled in a 2 ml collection tube containing MicroBead Solution (MO BIO Laboratories, Carlsbad, CA). The swab sponge was pressed against the wall of tube multiple times to ensure transfer of bacteria from the swab to the solution. Subsequently, swabs were given temperature shock twice at 90 °C in a water bath for 20 minutes and then at −80 °C to kill the bacteria. DNA purity was accessed by absorbance at 260 nm using a NanoDrop 1000 Spectrophotometer (Thermo Fischer Scientific, USA). The extracted DNA was stored at −20 °C until further processing.

### V1-V3 hyper-variable regions PCR amplification and sequencing

Amplification of the V1-V3 hyper-variable regions was performed using the bacterial 16S rRNA gene region specific primers. It was amplified using touchdown PCR with the conserved primer set 27F (5′–AGAGTTTGATCCTGGCTCAG-3′) and 518R (5′–GTATTACCGCGGCTGCTGG-3′). The amplicons were gel purified and then quantified by absorbance at 260 nm using a NanoDrop 1000 Spectrophotometer (Thermo Fischer Scientific, USA). DNA purity was assessed using the A260/A280 ratio. Samples failing amplification were amplified again using fresh aliquot until a satisfactory PCR product (for Illumina sequencing) was yielded. All the PCR amplicons were aliquoted and outsourced to DNA sequencing facility at Sandor Lifesciences Pvt. Ltd, Hyderabad, India. Sequencing was performed using the MiSeq platform (Illumina, Inc, San Diego, CA, USA) according to manufacturer’s instructions.

### Bioinformatics analysis

Total samples collected in this study were 90, however given the read quality issues with one of the unaffected region samples (Sample **A9**), paired exclusion was performed for Sample **A9** and its associated sample from the same participant i.e. Sample **G7**. Consequently, a total of 88 skin swab samples corresponding to (29) leprosy subjects and (30) controls (from Hyderabad and Miraj region of India) were used for the analysis. Table 1 provides an overview of the number of subjects and samples collected from the two mentioned research locations.

**Table 1:** An overview of the number of study participants and samples collected from the two-research locations viz. Hyderabad and Miraj.

Participant Type	Hyderabad		Miraj		Total Samples
Healthy (Control)	15		15		30
Patients	**Affected (Lesions)**	**Unaffected**	**Affected (Lesions)**	**Unaffected**	60
	14	14	16	16	

Data records file titled, ‘Metadata for Leprosy Microbiome Study’ provides a comprehensive metadata information table corresponding to all collected samples.

### Pre-processing

Raw reads of paired-end sequence data was subjected to standardized quality processing. High quality pairs with a minimum phred33 quality score of 25 were retained using prinseq v0.20.4^43^. Assembly of high quality pairs was performed using PEAR v0.9.10^44^ with a minimum overlap threshold of 10%. Only those reads were retained which had a minimum merged length of 400 bp. As mentioned above, given the poor quality of reads, one sample (Sample ID: A9 from an unaffected area of a study participant having leprosy) and its corresponding sample (taken from the affected region (G7) of the same participant) was discarded during the analysis process.

### Taxonomic classification

The quality checked and assembled sequence data was then subjected to closed reference classification using RDP classifier v.2.12^45^ at 0.8 minimum assignment confidence thresholds. Abundance data matrices at all five major levels of taxonomic lineage i.e. Phylum, Class, Order, Family and Genus were compiled using the hier files of RDP classification output (See section: Data Records). The sequence data was also processed using a de-novo OTU classification approach at a clustering identity of 0.97. The program vsearch v2.8.0_linux_x86_64^46^ was used as per the default protocol mentioned (for chimera removal and OTU clustering) at https://github.com/torognes/vsearch/wiki/VSEARCH-pipeline. Chimera removal was done using both protocols viz. mapping against the Gold reference database as well as the *de novo* chimera detection steps mentioned in the vsearch pipeline.

### Rarefaction

**Fig. 2:**
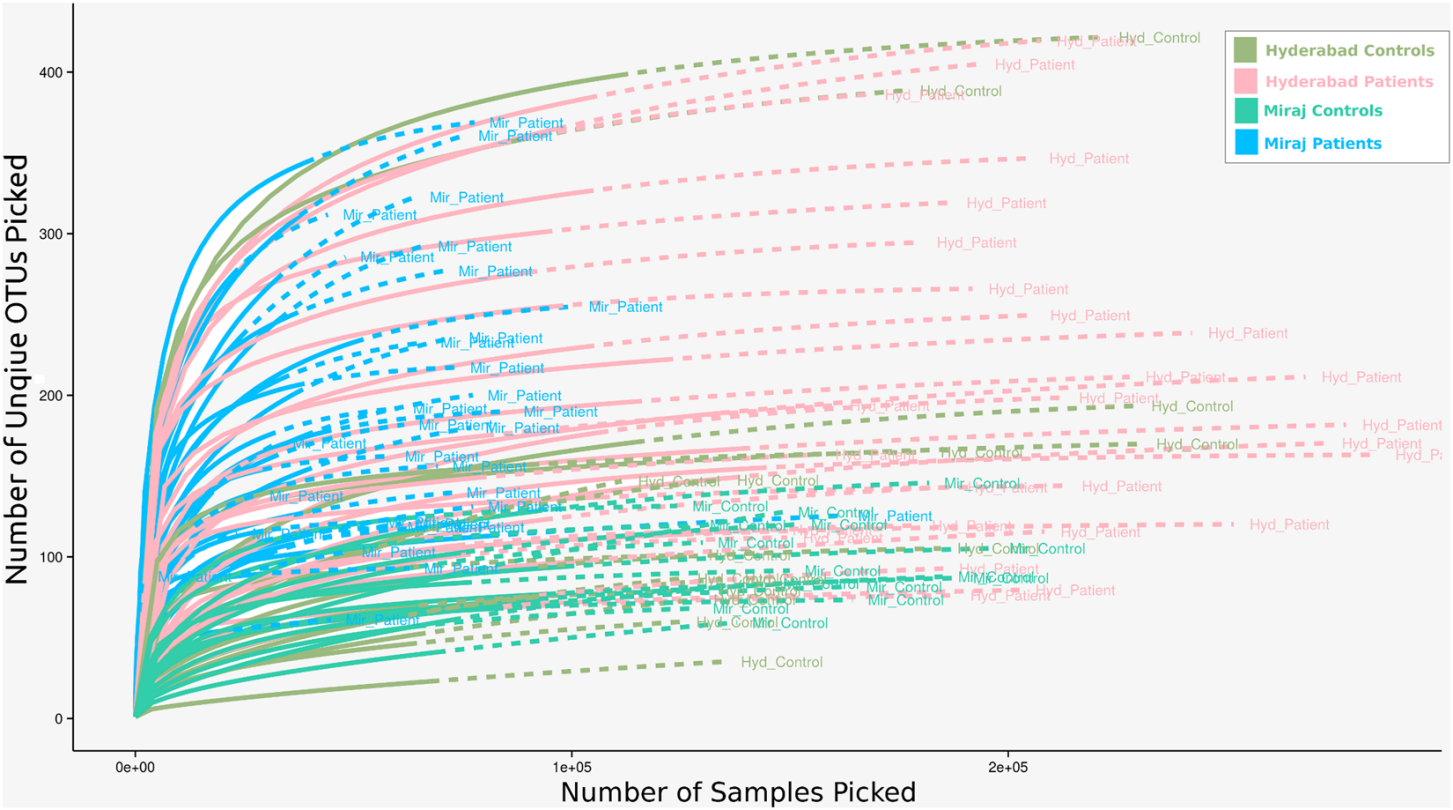
Rarefaction curves indicating sequencing depth for the samples collected in this study.

Rarefaction curves were plotted for the genus level abundance data using the observed species index generated by iNEXT v2.0.9^47^ (at 50 bootstrap replications for a knot size of 40 and end point equal to double the sample size) and ggplot2 v2.1.0.1^48^ R packages. Except for a few samples from Miraj, results of rarefaction depicted in Fig. 2 indicated sufficient sequencing depth for majority of the analyzed samples. The range of sample sizes for RDP assigned genus level OTUs was observed to be 543–1,31,889, with minimum sample size for Sample B2_16B and maximum size for G8_S15.

### Preliminary taxonomic assessment

Raw RDP taxonomic profiles generated indicated the presence of 1016 unique genera corresponding to 235 families, 90 orders, 55 classes and 25 phyla. Similarly, de novo OTU clustering generated a total of 161,695 OTUs. Two taxonomic abundance tables were generated (Refer section: Data Records). While the first table comprises taxonomic abundances of all 161,695 OTUs, the second table contains classification results for only those OTUs (28,321), which had a cumulative raw abundance value of two or more in the entire sample set (i.e. all population level singleton OTUs were discarded). Since the latter table has been filtered as per the mentioned criterion, we have provided the raw abundance table as well to provide researchers the flexibility to filter the same using any other criteria of their choice (Refer section: Data Records).

**Fig. 3:**
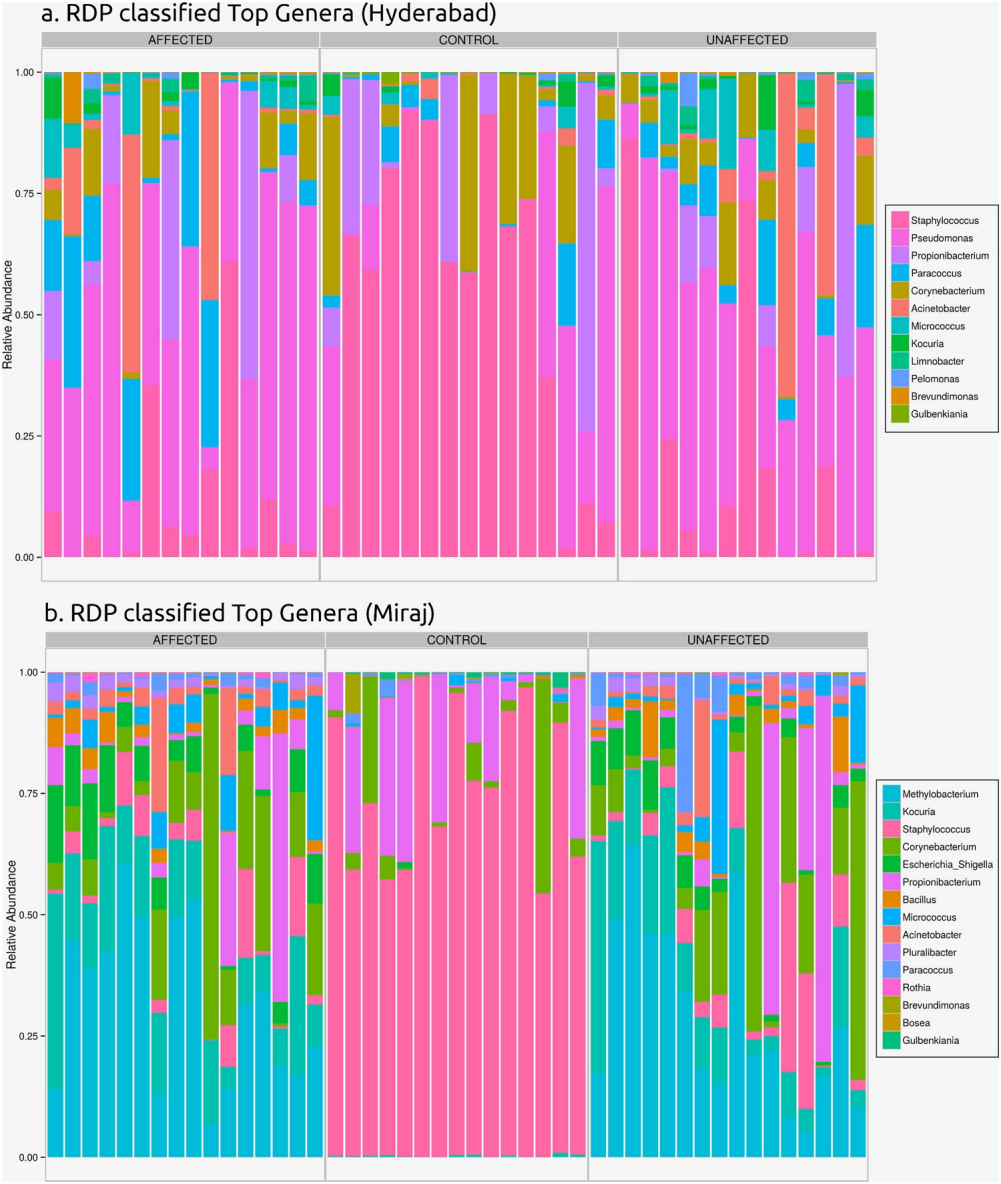
Relative abundance of top (RDP classified) genera in samples from study participants belonging to Hyderabad and Miraj locations.

**Fig. 4:**
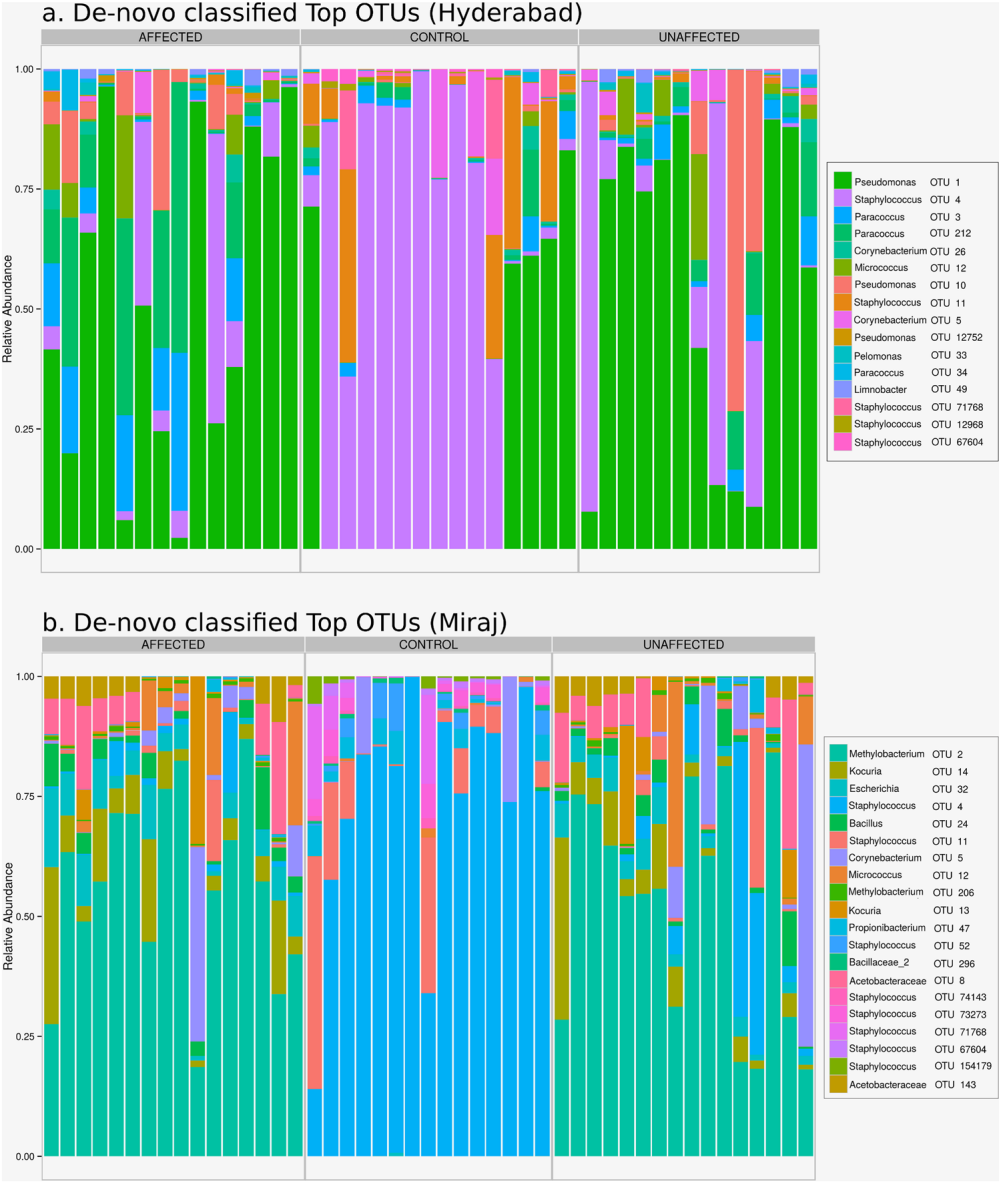
Relative abundance of top (RDP classified) de-novo generated OTUs (using vsearch) in samples from study participants belonging to Hyderabad and Miraj locations.

Figure 3a,b depict the relative abundance of top (RDP classified) genera in samples from study participants belonging to Hyderabad and Miraj respectively. Figure 4a,b depict the relative abundance of top (RDP classified) de novo generated OTUs in samples from study participants belonging to Hyderabad and Miraj respectively.

Preliminary analysis of taxonomic trends indicates the following three interesting trends.

1. In both sets of samples (belonging to Hyderabad and Miraj), a stark difference is observed between the taxonomic profiles corresponding to skin microbiome samples of healthy controls as compared to that from participants affected with leprosy. Figures indicate a distinct depletion of *Staphylococcus* in samples taken from leprosy affected participants.
2. There appears to be uniformity in skin microbiome profiles of healthy controls irrespective of geographical location (Hyderabad and Miraj). In contrast, the skin microbiome profiles of samples taken from leprosy affected participants from Hyderabad appear to have significant differences from that taken from affected participants from Miraj.
3. Absence of a distinct difference between samples taken directly from the leprosy lesional sites and those taken from adjoining unaffected regions.
4. Data record titled ‘Dendrogram Embedded Heatmap’ provides an overview of the relative compositional profile of skin microbiome across various classes of samples collected in this study. The heatmap was obtained by using Microbiomeanalyst^49^ at a prevalence threshold of one hit in at-least 20% of samples of the entire dataset. A centered log ratio transformation (CLR) of data was performed for the generation of heatmap. Euclidean distance was employed for generating OTU dendrogram embedded in the plot.

### Ordination

**Fig. 5:**
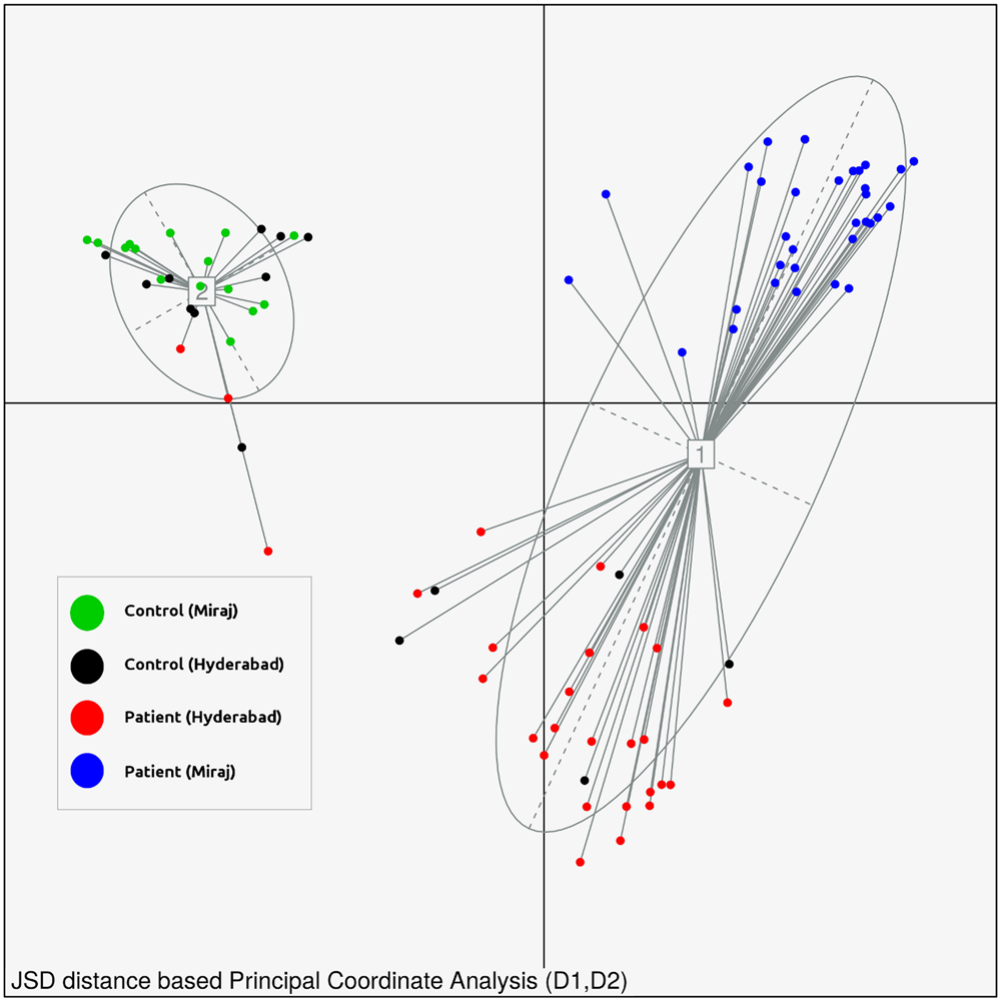
JSD based PCoA (Ordination) plot for all samples of the study, colored by the nature (control or patient) and geographical affiliation (Miraj or Hyderabad) of samples.

In order to obtain preliminary insights into the compositional similarities (and differences) between samples pertaining to controls and patients, as well as the impact of different geographical affiliations, Jansen-Shannon divergence (JSD) and Partitioning Around Medoids (PAM) based PCoA was employed^27,50^. Figure 5 represents the ordination analysis performed using all samples in this study with meta-data affiliation pertaining to both state of health of participants and geography of sampling. It was interesting to observe clustering together of control samples irrespective of their geographical affiliation. In addition, geography specific segregation of samples pertaining to patients from Miraj and Hyderabad centers was apparent. Data record titled, ‘Dendrogram Embedded Heatmap’ (as introduced earlier) further depicts a dendrogram embedded heatmap based on taxa abundances in each of the samples of the entire study. Given the preliminary nature of these observations, a detailed analysis of the depicted pattern and assessment of microbiome characteristics with respect to its community diversity (alpha and beta) characteristics, associations or correlations between microbial abundances and associated metadata, inferred functional analysis using tools like Picrust^51^ and/or iVikodak^52^ is beyond the scope of the current article. However, we anticipate that the data we have provided along with this report will allow researchers to investigate and compare the provided skin microbiome profiles with other available analogous datasets.

## Data Records

We share three data types corresponding to the present study. We have made sincere attempts to organize the data in a manner that makes it amenable for further analysis. While Data type 1 (i.e 16S rDNA sequence data) is available for download at the NCBI Sequence Read Archive (Study: SRP187334^53^), the other two data types (i.e. Metadata and Taxonomic abundance datasets^54^) have been provided as a part of this publication (accessible through figshare).

### FASTQ data

The sequencing data corresponding to all 90 samples are accessible at NCBI Sequence Read Archive (SRA) as BioProject: PRJNA505133 (Study: SRP187334^53^); Title: 16S rDNA based skin bacterial flora from leprosy patients and healthy individuals using Illumina MiSeq sequencing. We have shared fastq data to enable re-analysis by end-users who intend/ prefer to employ customized computational protocols and/ or parameters of their choice.

### Study metadata

In addition to microbiome data corresponding to sampled skin sites, the study additionally recorded Age, Sex, Disease Spectrum and Diagnosis record as aspects of the participating subjects. This is provided as a tab-separated file titled, ‘Metadata for Leprosy Microbiome Study’ that would facilitate customized data analysis in future retrospective studies.

### Taxonomic abundance tables

As mentioned previously, we provide RDP classified taxonomic abundance profiles^54^ at all five levels of taxonomic lineage and two distinct de-novo classification based taxonomic abundance tables. RDP classified datasets may be accessed through the data files titled:

1. Leprosy Microbiome **Genus** Level Abundance Data
2. Leprosy Microbiome **Family** Level Abundance Data
3. Leprosy Microbiome **Order** Level Abundance Data
4. Leprosy Microbiome **Class** Level Abundance Data
5. Leprosy Microbiome **Phylum** Level Abundance Data

Data files corresponding to the de-novo classified OTUs may be accessed through the files titled:

1. Leprosy Microbiome OTU Level Abundance Data **(Unfiltered, raw set)**
2. Leprosy Microbiome OTU Level Abundance Data **(Filtered Set)**

Data file corresponding to the image representing (Swab) sample collection process may be accessed through the file titled:

1. Sample Collection Image

Data file corresponding to the figure representing a dendrogram embedded heatmap based on genus level taxonomic abundances in all samples of the study may be accessed through the file titled:

1. Dendrogram Embedded Heatmap

## Technical Validation

Ethical clearances were obtained from IEC, NCCS and leprosy clinics for performing experiments and implementing quality testing for the study. All the informed consents and questionnaire for counseling were precisely documented. The required questionnaire was bilingual in nature. Each individual enrolled in the study was rehearsed with the complete study design and protocols.

### Sample management

Data entry was done in compliance with the data records obtained from the leprosy clinics. Multiple checks were done regularly to ensure its authenticity and validity.

### DNA sample and PCR amplification quality control

The amount of DNA extracted from skin swabs was in extremely low amounts. Therefore, an aliquot of DNA extracted from each swab was used for PCR amplification using Touch-down protocol. PCR was performed in a Laminar flow cabinet after overnight UV illumination. The amplified product was then quantified using NanoDrop^®^ ND-1000 UV-Vis Spectrophotometer (Thermo Fischer Scientific, USA) and checked for its quality using agarose gel electrophoresis. The PCR product was further outsourced for the amplicon library preparation and sequencing.

### Sequence quality assessment and bioinformatics pipeline

Out of the total 85,57,782 good quality reads employed for reference based taxonomic classification, only 6,08,313 (~7%) remained unclassified. A fairly good percentage (55,38,880 i.e. ~65%) of the good quality reads were assigned to the most specific (genus) level using RDP classifier. In addition, a total of 161,695 OTUs were identified using vsearch based de-novo classification, which were filtered to 28,321 unique OTUs by omitting the population-level singletons.
